# ArpA-like regulatory control and discovery of new biosynthetic genes driving scleric acid biosynthesis

**DOI:** 10.1039/d6cb00137h

**Published:** 2026-06-26

**Authors:** Jingfan Zhang, Lijiang Song, Manuela Tosin, Christophe Corre, Fabrizio Alberti

**Affiliations:** a School of Life Sciences, University of Warwick Coventry CV4 7AL UK C.Corre@warwick.ac.uk F.Alberti@warwick.ac.uk; b Department of Chemistry, University of Warwick Coventry CV4 7AL UK

## Abstract

Scleric acid is a natural product that was discovered by heterologous expression of the silent *scl* gene cluster from *Streptomyces sclerotialus* in *Streptomyces albidoflavus* and rational de-repression of the biosynthetic genes through deletion of the ArpA-like regulator SclM4. It is a weak inhibitor of the cancer-associated enzyme nicotinamide *N*-methyltransferase (NNMT). In this work we aimed to improve understanding of scleric acid biosynthesis and of gene regulation of the *scl* gene cluster. We performed CRISPR/Cas9-mediated deletion of the biosynthetic gene *sclG*, which confirmed the essential role of the corresponding ATP-grasp family enzyme in the biosynthesis of scleric acid. We also carried out whole-genome Nanopore long-read sequencing of *S. sclerotialus* to investigate the borders of the *scl* gene cluster, which led us to discover two additional genes, *sclC* and *sclK*, proposed to be involved in biosynthetic precursor supply. Transcriptomics analyses were performed to investigate the effect of the deletion of the ArpA-like gene *sclM4* on the expression of the *scl* biosynthetic genes, shedding light on the role of the corresponding transcriptional repressor. The present work improves understanding of scleric acid biosynthesis and provides new insights into the ArpA-like mediated regulation of biosynthetic gene expression in *Streptomyces* bacteria.

## Abstract 2

Characterisation of the *scl* biosynthetic gene cluster reveals ArpA-like regulation of gene expression and identifies new genes involved in scleric acid biosynthesis and precursor supply.

## Graphical abstract

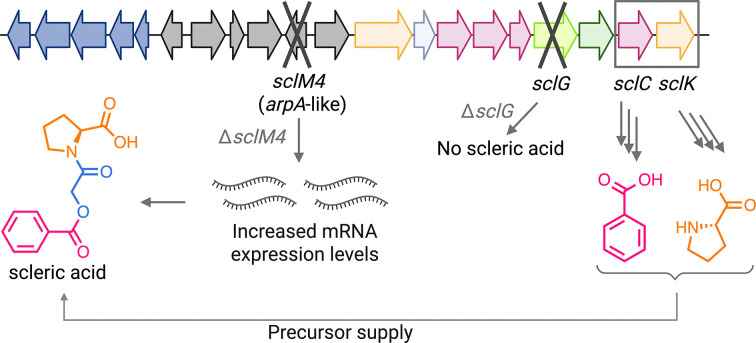

## Introduction

Secondary metabolites display enormous structural diversity, but the biosynthetic principles for many of them are highly conserved.^1^ As a result, within each biosynthetic class (*e.g.* polyketide synthases (PKSs), nonribosomal peptide synthetases (NRPSs), ribosomally synthesised and post-translationally modified peptides (RiPPs)), many of the core and tailoring enzymes have highly conserved amino acid sequences.^1,2^ This significant sequence similarity facilitates their identification based solely on nucleotide sequences, enabling easier prediction and annotation of biosynthetic pathways.

**Fig. 1:**
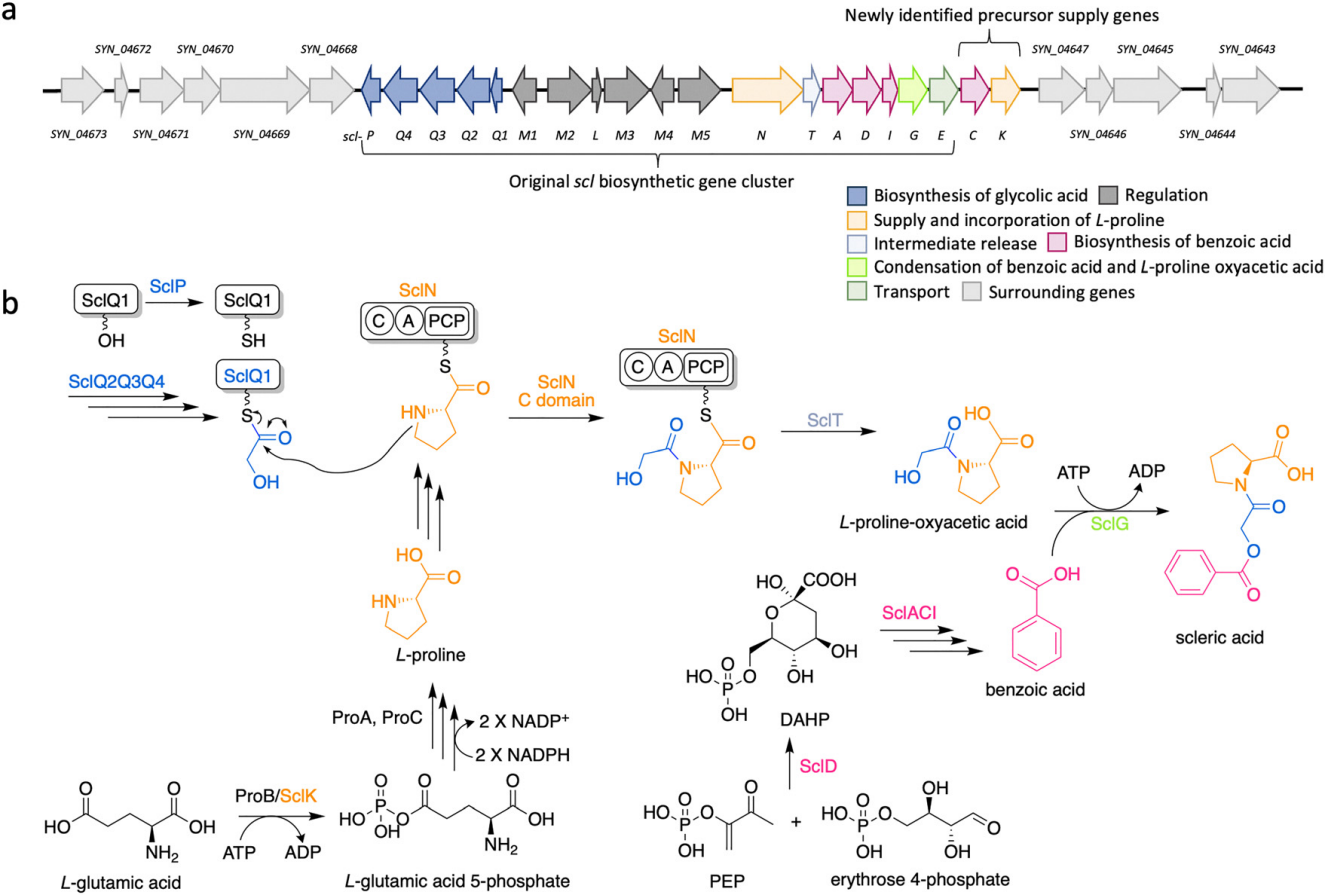
(a) *Scl* gene cluster identified in ref. 3. The original cluster is 19 782 bp in size and comprises of 11 biosynthetic genes (*sclQ1-P* and *sclN-G*), 6 genes for regulation (*sclM1-5* are homologous to *mmyR-mmfR-mmfLHP*; *sclL* is homologous to the LysR-family transcriptional regulator) and 1 transporter (*sclE*). Two novel genes, *sclC* and *sclK*, predicted to be part of the *scl* gene cluster are included. Surrounding genes detected by antiSMASH are also included although not believed to take part in scleric acid biosynthesis. The proposed functions of the proteins encoded by genes from the *scl* gene cluster can be found in ref. 3. (b) The proposed biosynthetic pathway of scleric acid, including proposed roles for the newly identified genes *sclC* and *sclK* in precursor supply. PEP = phosphoenolpyruvic acid; DAHP = 3-deoxy-d-arabino-heptulosonate 7-phosphate.

The *scl* gene cluster was originally discovered in *Streptomyces sclerotialus* NRRL ISP-5269 through a multigene BLAST and ClusterTools search for homologous sequences of the *mmyR-mmfR-mmfLHP* methylenomycin (Mm) regulatory cassette from *Streptomyces coelicolor* A3.^3^ MmyR and MmfR are both paralogues of ArpA with 32% amino acid identity, whereas MmfL is homologous to AfsA. However, the strain containing only MmfL lacks the ability to produce methylenomycin furan (MMF) signalling molecules, indicating that MmfH and MmfP are also significant in the biosynthesis of MMFs.^4,5^ This was confirmed by further mutagenesis analysis.^4^ The *scl* cluster contains orthologues of all five genes as well as an additional LysR-family transcriptional regulator and two divergent sets of biosynthetic genes (Fig. 1a).^3^

The product of the *scl* gene cluster was characterised to be the novel molecule scleric acid, which was discovered using a combination of transformation-associated recombination (TAR) cloning and rational de-repression of the *scl* gene cluster by *sclM4* knockout. Scleric acid showed moderate antibacterial activity against *Mycobacterium tuberculosis* (H37Rv) and inhibitory activity against the cancer-associated enzyme nicotinamide *N*-methyltransferase (NNMT) with IC_50_ of 178.0 µM (1-methylnicotinamide (MNAN) product detection) and 186.6 µM (*S*-adenosylhomocysteine (SAH) co-product detection).^3^ The two IC_50_ values are within 5% of each other, indicating that scleric acid inhibits NNMT consistently, regardless of which reaction product is being monitored.

The involvement of *sclQ1-4*, *sclN* and *sclA* in scleric acid biosynthesis was confirmed through additional rounds of CRISPR/Cas9-mediated knockouts, alongside detection of the intermediate l-proline-oxyacetic acid in selected strains, which led to a proposed biosynthetic pathway (Fig. 1b).^3^ Three main building blocks were identified: a glycolic acid unit biosynthesised by SclQ1-4, an l-proline residue activated by SclN, which carries out a condensation reaction with glycolic acid, a benzoic acid unit biosynthesised by SclADI. The benzoic acid unit is proposed to be activated by SclG and a condensation reaction between activated benzoic acid and l-proline-oxyacetic acid is supposed to be catalysed by SclG to form scleric acid.^3^

In the present study, we aimed to improve understanding of scleric acid biosynthesis and regulation. Specifically, we proved that the ATP-grasp enzyme SclG has an essential role in scleric acid biosynthesis. We also identified the borders of the *scl* gene cluster through Nanopore whole-genome sequencing, which complemented the Illumina-based sequencing previously made available for *S. sclerotialus*. Lastly, we investigated the regulatory mechanism underlying scleric acid biosynthesis through the deletion of the transcriptional regulator *sclM4*, and investigated its effect on transcription of the scleric acid biosynthetic genes.

## Results

### The ATP-grasp family enzyme SclG is involved in scleric acid biosynthesis

Firstly, we set out to investigate the involvement of the ATP-grasp family enzyme SclG in scleric acid biosynthesis, given its key role in the proposed pathway to scleric acid (Fig. 1b). We performed a knockout of *sclG* in the scleric acid producing strain, *S. albidoflavus*/*scl* Δ*sclM4*, to complement those already generated on the other biosynthetic genes *sclA*, *sclN* and *sclQ1-4*^3^ and thus provide a full picture of the biosynthetic pathway to scleric acid.

**Fig. 2:**
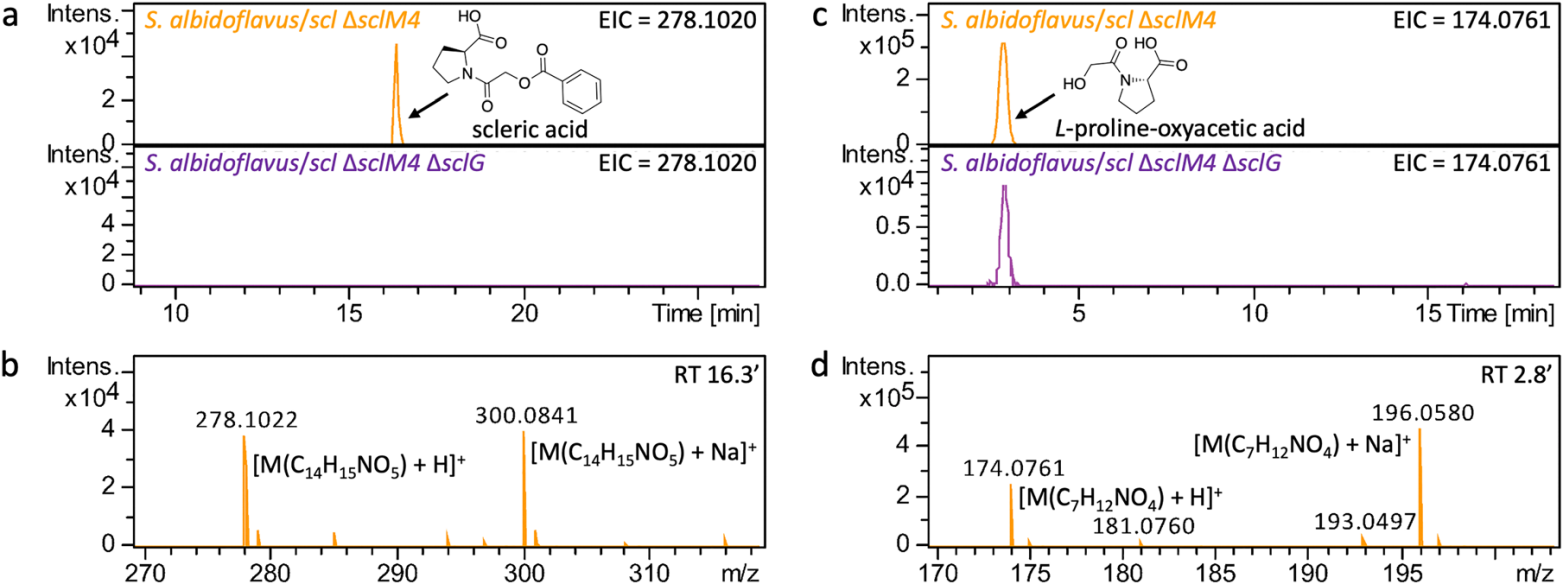
LC-HRMS detection of metabolites produced in *S. albidoflavus*/*scl* Δ*sclM4* (orange) and *S. albidoflavus*/*scl* Δs*clM4*Δs*clG* (purple). (a) Extracted ion chromatograms (EICs) in positive mode for *m*/*z* 278.1020 are shown highlighting scleric acid accumulation in *S. albidoflavus*/*scl* Δ*sclM4* but not in *S. albidoflavus*/*scl* Δs*clM4*Δs*clG*. (b) Mass spectrum in positive mode of scleric acid. (c) EICs in positive mode for *m*/*z* 174.0761 are shown highlighting l-proline-oxyacetic acid accumulation in both *S. albidoflavus*/*scl* Δ*sclM4* and *S. albidoflavus*/*scl* Δs*clM4*Δs*clG*. (d) Mass spectrum in positive mode of l-proline-oxyacetic acid.

Plasmid pCRISPomyces-2 (pCm2) was used for this purpose, aiming to delete the entire *sclG* sequence.^6^ The sgRNA sequence was designed (Table S1), alongside homologous recombination arms of 800 bp to flank the region to be deleted. Strain *S. albidoflavus*/*scl* Δs*clM4*Δs*clG* was generated (Fig. S1) and metabolite extracts were analysed in comparison to those of strain *S. albidoflavus*/*scl* Δs*clM4*. The deletion of *sclG* abolished production of scleric acid, while production of the intermediate l-proline-oxyacetic acid was retained (Fig. 2), in accordance with the proposed function of SclG to be involved only in the last biosynthetic step of scleric acid production (Fig. 1b).

### Nanopore long-read genome sequencing of *S. sclerotialus* NRRL ISP-5269 and biosynthetic gene cluster analysis

The only genome data available for *S. sclerotialus* NRRL ISP-5269 prior to the present study was from an Illumina sequencing project (GenBank accession number JOBC01000043.1) that assembled it in 92 contigs. Importantly for this study, the contig that contains the *scl* gene cluster ends with the transporter *sclE*, raising questions regarding the true border of the *scl* cluster. Thus, Nanopore sequencing was carried out here to provide a more robust genome assembly of *S. sclerotialus*.

A high-quality assembled and polished genome was produced, resulting in two contigs, and with a BUSCO score of 99.5% (Fig. S2 and S3). The genome was deposited at DDBJ/ENA/GenBank under the accession JBFOHP000000000. Annotations of the assembled and polished *S. sclerotialus* NRRL ISP-5269 genome against the Nucleotide collection (nr/nt) database were performed for the general features of the chromosome sequence (Table S2).

Using antiSMASH bacterial version 8.0.4,^7,8^ we were able to identify 36 biosynthetic gene clusters (BGCs) for secondary metabolites in the newly assembled *S. sclerotialus* NRRL ISP-5269 genome, including the *scl* BGC (Fig. S4 and Table S3). Nearly 1/3 of the *S. sclerotialus* NRRL ISP-5269 clusters are predicted to produce antibiotic-like molecules. Most of the BGCs are predicted to encode for NRPS, PKS or NRPS/PKS hybrids, followed by terpene synthases, RiPPs and siderophores. It is interesting that four BGCs are proposed to encode for siderophore biosynthesis, which implies that *S. sclerotialus* NRRL ISP-5269 is likely to undergo strong selective pressure in low iron environments.^9^ There are also a few regions containing ectoine, alkaloid and saccharide type BGCs. In conclusion, *S. sclerotialus* NRRL ISP-5269 has the potential to produce a plethora of natural products with different biological functions that are still to be explored.

### Border definition of the *scl* gene cluster and proposed role of *sclC* and *sclK*

The *scl* BGC identified from the antiSMASH analysis of the assembled and polished genome sequence revealed the presence of additional genes outside of the original *scl* gene cluster characterised in ref. 3 (Fig. 1a). Genes adjacent to the *scl* BGC were further investigated, and their amino acid sequences were analysed through BlastP for homology and putative function investigation (Table S4).^10^ Two genes downstream of *sclE* – *sclC* and *sclK* – were predicted to be part of the *scl* gene cluster and contribute to the biosynthesis of scleric acid precursors based on their putative functions (Tables S5 and S6).

SclC is proposed to be a chorismate synthase involved in the biosynthesis of benzoic acid, along with SclADI. SclA is a proposed anthranilate synthase, SclD is a proposed DAHP synthase and SclI is a proposed isochorismatase.^3^ These four enzymes are proposed to work closely together to provide benzoic acid (Fig. 1b).^11–13^

One of the main microbial pathways to produce l-proline is through sequential catalysis by ProB (glutamate 5-kinase), ProA (glutamate 5-phosphate reductase) and ProC (pyrroline-5-carboxylate reductase).^14,15^ Within the *S. sclerotialus* NRRL ISP-5269 genome, two genes were found to be orthologues of *proA*, and two genes were found to be orthologues of *proB*, including *sclK* within the *scl* gene cluster, and one gene was found to be an orthologue of *proC*. This further suggests that l-proline is produced in *S. sclerotialus* NRRL ISP-5269 *via* this pathway and that SclK is likely to be part of the *scl* gene cluster and to be involved in the biosynthesis of l-proline that is then used as a building block for scleric acid (Fig. 1b).

*S. albidoflavus*/*scl* Δ*sclM4* appeared to be able to produce scleric acid without the need for *sclC* and *sclK*, since these two genes were not included in the original heterologously-expressed *scl* cluster. This is likely due to the expression of chorismate synthase and glutamate 5-kinase in the heterologous host *S. albidoflavus*, which complement the lack of these two genes in the *scl* heterologously expressed gene cluster.^3^ Indeed, BlastP searches confirmed the presence of *sclC* and *sclK* homologues in the *Streptomyces albidoflavus* genome (Table S6).

### Knockout of *sclM4* leads to transcriptional changes in the *scl* gene cluster

Transcriptional changes in the *scl* gene cluster following inactivation of *sclM4 via* a 20-bp deletion were investigated to assess how the biosynthetic genes and the regulatory genes are controlled by SclM4. The newly discovered genes *sclC* and *sclK* were not included in the transcriptional study due to their absence from the plasmid originally integrated in *S. albidoflavus*. Transcript coverage analysis was also performed to find the promoter region(s) and thus the operon structure of the *scl* gene cluster.

**Fig. 3:**
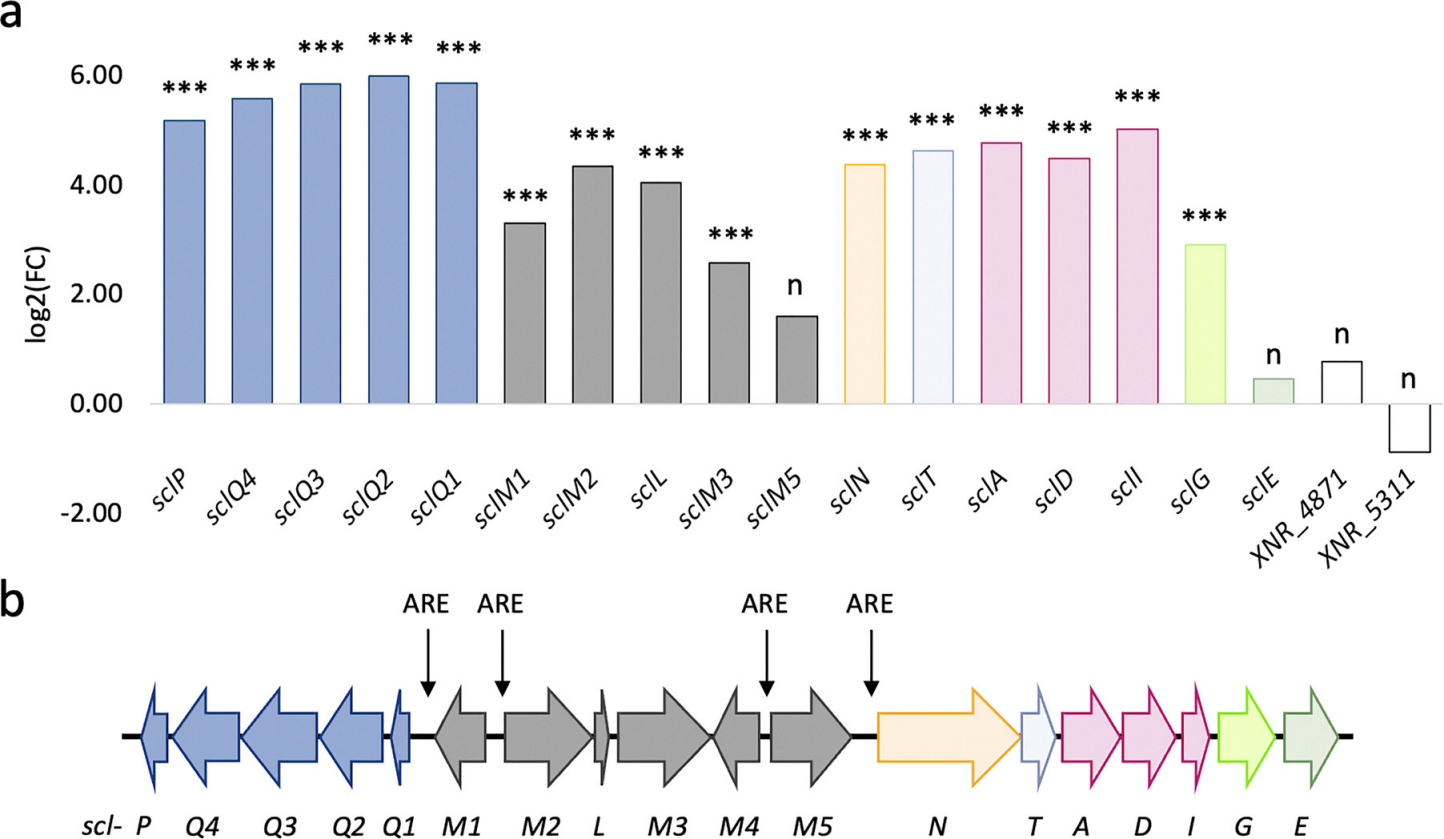
(a) Log 2(FoldChange(FC)) of normalised gene counts from *S. albidoflavus*/*scl* Δ*sclM4* compared to normalised gene counts from *S. albidoflavus*/*scl*. The biosynthetic genes (*sclP-Q1*, *sclN-G*) were upregulated upon deletion of *sclM4*. *XNR-4871* and *XNR_5311* are two reference genes used for comparison. The log 2(FC) of the two reference genes between *S. albidoflavus*/*scl* Δ*sclM4* and *S. albidoflavus*/*scl* have small *P*-adj but their |log 2(FC)| falls within the cutoff of 2, therefore the transcription does not change significantly. Precise normalised counts, log 2(FC) value, *P*-value and *P*-adj can be found in Tables S7 and S8. *P*-adj higher than 0.05 is marked with (*n*). *P*-adj lower than 0.001 is flagged with (***). *n* = 3. (b) The *scl* biosynthetic gene cluster heterologously expressed in *S. albidoflavus*/*scl*. The arrows point to the proposed autoregulatory elements (AREs) in the gene cluster, which are locations proposed to be ArpA-family regulator binding sequences.

RNA sequencing of *S. albidoflavus*/*scl* Δ*sclM4* and *S. albidoflavus*/*scl* was performed and the normalised counts were collected, compared (see Tables S7 and S8 for the comparison of the genes belonging to the *scl* gene cluster) and visualised (Fig. S5 and Fig. 3). Upon knocking out *sclM4*, all the genes taking part in the biosynthetic pathway of scleric acid appeared to be upregulated, which is in agreement with the observed production of scleric acid in *S. albidoflavus*/*scl* Δ*sclM4*.^3^

In contrast, *sclM5* and *sclE* did not appear to be affected by the deletion of *sclM4*. The Integrative Genomics Viewer (IGV) visualization of *sclM5* suggests that the RNAseq reads available for SclM5 do not cover the full gene sequence in both the *S. albidoflavus*/*scl* Δ*sclM4* strain and the *S. albidoflavus*/*scl* strain (Fig. S6). This could be a pseudo-truncation arising from sequencing artifacts associated with high-GC RNA. The lack of upregulation of *sclM5* in the *S. albidoflavus*/*scl* Δ*sclM4* strain may be due to the 20-bp deletion of *sclM4* that is present in this strain, which can be observed from the visualised transcriptome map of *sclM4* (Fig. S7). The 20-bp deletion may be responsible for decreasing *sclM5* gene expression driven by the promoter located upstream of *sclM2*, which is known to direct the expression of the whole *mmfLHP* regulatory cassette in *S. coelicolor*.^4^

The transporter *sclE* belongs to the major facilitator superfamily (MFS), which uses the proton-motive force in the transport process.^16^ The expression levels of *sclE* remained consistently high both before and after the deletion of *sclM4* (Table S7). Thus, it is possible that it is not affected by SclM4, yet it can still contribute to scleric acid secretion.

SclM1 is homologous to the *S. coelicolor* MmfR (56% identity/73% similarity) and SclM4 is homologous to MmyR (41% identity/56% similarity). MmfR and MmyR are well studied ArpA-family repressors that were confirmed to each act as a repressor. Each of MmfR and MmyR homodimers are proposed to bind independently to the same DNA motif, the MARE (MMF-autoregulator responsive elements) sequence.^4,17,18^ However, only MmfR is sensitive to the signalling molecule MMF.^4^ Due to the homology of SclM1/MmfR and SclM4/MmyR, SclM1 and SclM4 are hypothesised to bind to the same ARE motif. Operon determination in *Streptomyces* is complicated by the prevalence of leaderless transcripts. Nonetheless, by focusing on intergenic regions which are present regardless of transcript architecture, multiple expectation maximizations for motif elicitation (MEME) enrichment analysis was performed on the *scl* gene cluster to identify the proposed ArpA-family repressor binding site for SclM1 and SclM4 (Fig. 3b and Table S9).^18^ The location of the four ARE sites and the six associated start codons are listed (Table S9). Promoter regions near these ARE motifs were all confirmed by visualization of the RNA sequencing reads on IGV (Fig. S8), further confirming the proposed SclM4 binding sites.

## Discussion

*Streptomyces* are one of the most important sources of medicines and agrochemicals due to their ability to produce a wealth of bioactive natural products, such as antibiotics, herbicides, anti-cancer agents and immunosuppressants, through complex secondary metabolic biosynthesis pathways.^9^ In this study, we set out to investigate the biosynthesis of scleric acid and the regulation of its biosynthetic gene expression. The involvement of SclG in scleric acid biosynthesis was investigated. SclG is proposed to be an ATP-grasp domain-containing enzyme that activates benzoic acid and catalyses a condensation reaction between the activated benzoic acid and l-proline-oxyacetic acid to form scleric acid.^3^ In this study, *sclG* was knocked out from the scleric acid producing strain *S. albidoflavus*/*scl* Δ*sclM4*, leading to production of scleric acid to cease. l-Proline-oxyacetic acid was still detected from the metabolite extracts of the *S. albidoflavus*/*scl* Δ*sclM4* Δ*sclG* strain, indicating that SclG does not take part in the biosynthesis of l-proline-oxyacetic acid but is needed in the last step of scleric acid biosynthesis.

Prior to the present work, the genome of *S. sclerotialus* NRRL ISP-5269 had been sequenced using Illumina technology, resulting in 92 contigs. In this work, the genome of *S. sclerotialus* was sequenced *via* Nanopore long-read technology, resulting in 2 contigs and a complete chromosome. Two genes, *sclC* (encoding a chorismate synthase) and *sclK* (encoding a glutamate 5-kinase), adjacent to *sclE*, were found to likely be part of the scleric acid BGC. They are proposed to be involved in the biosynthesis of building blocks (benzoic acid and l-proline, respectively) of scleric acid, and their absence in the scleric acid-producing heterologous strain^3^ was proposed to be complemented by genes with homologous functions present in the *S. albidoflavus* genome. Chorismate synthase and glutamate 5-kinase are typically associated with primary metabolism. Chorismate is often the obligatory precursor for benzoic acid, and housing a chorismate synthase (*i.e.* SclC) within the BGC would ensure a localised supply of chorismate for benzoic acid production. Glutamate 5-kinase (SclK) is proposed to catalyse the first committed step in l-proline biosynthesis. The duplication of primary metabolic enzymes into BGCs ensures the precursor supply and enables pathway self-sufficiency for secondary metabolism, and also helps maintain cell functionality during stress such as secondary metabolites production or nutrient limitation.^19^

We next turned our attention to the regulatory cassette SclM1-5 from the scleric acid gene cluster, which is homologous to the well-studied regulatory cassette (MmfR/MmyR/MmLHP) of the methylenomycin biosynthetic gene cluster in *S. coelicolor*.^3^ The knockout of *mmyR* is known to trigger the production of Mm.^4^ However, no studies were performed on the effects of the deletion of MmyR, or of its homologue SclM4, on the transcription of the biosynthetic genes from their respective BGCs. In this study, comparative RNA sequencing analysis was carried out between *S. albidoflavus*/*scl* Δ*sclM4* and *S. albidoflavus*/*scl*. Generally, it is assumed that the disruption of the AfsA homologues (*e.g.* MmLHP and SclM2/3/5) leads to reduced or ceased production of the target natural products, whereas mutation of the ArpA homologues (*e.g.* MmyR/SclM4) leads to the opposite effect.^20^ For example, the inactivation of *afsA* homologues *shbA1*, *shbA2* or *shbA3* in *S. hygroscopicus* 5008 decreased the biosynthesis of validamycin whereas the deletion of *arpA* homologues *shbR1* or *shbR3* increased the production of validamycin.^21^ This hypothesis was confirmed in our study, where the knockout of *sclM4* led to the upregulation of *sclM2* and *sclM3*, and further resulted in the initiation of scleric acid production. However, exceptions have also been observed, such as the AfsA/ArpA-like system SbbA/SbbR in *S. bingchenggensi*s, in which the inactivation of the signal molecule synthase gene *sbbA* resulted in an increased production of the final secondary metabolite, whereas mutation of *sbbR* led to reduced production of the final product.^20^

It emerged that upon *sclM4* knockout, all the biosynthetic genes from the *scl* gene cluster were upregulated. The gene *sclM5*, involved in the biosynthesis of signalling molecules, was not significantly upregulated, which may be due to a biological sequencing error or to the upstream 20-bp deletion made within the *sclM4* sequence in *S. albidoflavus*/*scl* Δ*sclM4*^3^ affecting the expression of *sclM5*. The transporter *sclE* was also not upregulated, though still expressed at high levels in both strains.

## Conclusions

In this study, we improved understanding of scleric acid biosynthesis and of gene regulation in the *scl* gene cluster. We identified genes putatively involved in precursor supply at the edge of the *scl* gene cluster. We also showed that the gene cluster-specific ArpA-like transcriptional regulator can influence the expression of both scleric acid biosynthetic genes and signalling molecule biosynthesis genes.

## Materials and methods

### Culturing conditions

*Streptomyces* strains were grown in Soy Flour Mannitol (SFM) (20 g soya flour, 20 g mannitol, 20 g agar, tap water to a final volume of 1000 mL) at 30 °C.

### sgRNA design

The nucleotide sequence of *S. sclerotialus* NRRL ISP-5269 was uploaded onto the CRISPY-web tool.^22^ The CRISPY-web tool then generated sgRNAs based on the knockout's target gene, which included a 20 nt protospacer and a 3 nt PAM sequence. Because the *scl* gene cluster is heterologously expressed in *S. albidoflavus* J1074, the result from the CRISPY-web tool was manually aligned against the *S. albidoflavus* J1074 genome using BLAST, to avoid off-target effects on the host genome.

The sgRNA close to the centre of the target gene was chosen. Its DNA oligonucleotides were then ordered from Integrated DNA Technologies, with BbsI-compatible overhangs for the Golden Gate assembly (5′-ACGC for forward and 5′-AAAC for reverse).

### pCm2 plasmid construction

Construction of pCm2-sgRNA-HRs plasmid able to perform HDR knockouts *in vivo* involved two steps. Firstly, Golden Gate assembly was performed to insert the protospacer into the sgRNA cassette of the plasmid. Secondly, Gibson assembly was performed to insert the HR arms downstream of the sgRNA cassette.^23^ The linearisation of pCm2-sgRNA vectors for Gibson assembly was performed with XbaI (NEB).

### Intergeneric conjugation of plasmids from *E. coli* to *Streptomyces*

Plasmid was transformed into *E. coli* ET12567/pUZ8002 electrocompetent cells *via* electroporation. The *E. coli* ET12567/pUZ8002 plasmid mixtures were grown to an OD_600_ of 0.4. Whilst *E. coli* was growing, spores from the target *S. albidoflavus* strain were harvested and resuspended in 1 mL of 2× YT broth (16 g tryptone, 10 g yeast extract, 5 g NaCl, dH_2_O for a final volume of 1000 mL). They were preserved at 4 °C before the OD_600_ of *E. coli* reached 0.4. Once the OD_600_ of *E. coli* ET12567/pUZ8002 reached 0.4, they were harvested by centrifugation and washed in LB twice to remove residual antibiotics. The *E. coli* cells were then resuspended in 1 mL LB. When *E. coli* was ready, *Streptomyces* spores were heat shocked at 50 °C for 10 minutes for germination and left at room temperature to cool.

500 µL of *E. coli* ET12567/pUZ8002 cells containing the target plasmid and 500 µL of *Streptomyces* spores were mixed and centrifuged at 4000 rpm for 2 minutes. 900 µL of the supernatant was removed and the *Streptomyces*/*E. coli* mixture was resuspended in the remaining 100 µL by gently pipetting up and down. Both the ten-fold dilution and the neat mixture were then spread on SFM agar plates containing 10 mM MgCl_2_. After an overnight (16–20 hours) incubation at 30 °C, the conjugation plates were overlaid with 1 mL sterile solution of 0.5 mg nalidixic acid and 1 mg kanamycin. The plates were then incubated at 30 °C for 2–5 days until exconjugants appeared. Exconjugants were further cultured on SFM plates containing 50 µg mL^−1^ kanamycin and 25 µg mL^−1^ nalidixic acid. The plates were incubated for an additional 4–6 days until single colonies appeared. Single colonies were sub-cultured onto SFM plates containing 50 µg mL^−1^ kanamycin.

### Screening for clones containing the expected plasmid using colony PCR

Amplification of the target knock-out regions was performed using colony PCR. The primers used were pCm2-*sclG* forward and pCm2-*sclG* reverse (Table S10).

The PCR results were then analysed by gel electrophoresis in a 1% (w/v) agarose gel in 1X TAE buffer containing 1X GelRed. The gel was run at 100 V for 60 minutes. It was then visualized and analysed. Six of the potential knockout strains were then picked and their genomic DNA was extracted using MP Biomedicals™ FastDNA™ SPIN Kit for Soil. PCR amplification was carried out using primers pCm2-*sclG scl* FF and pCm2-*sclG scl* RR (Table S10). The PCR was then run using gel electrophoresis in a 1% (w/v) agarose gel in 1X TAE buffer containing 1X GelRed. The gel was run at 100 V for 60 minutes. Amplified gDNA fragments were extracted from the gel using a GeneJET Gel Extraction Kit. Deletion was then confirmed through Sanger sequencing.

### Metabolite extraction and analysis

*Streptomyces* strains were cultured on 33 mL of supplemented minimal media solid (SMMS), prepared according to Strauch *et al.*^24^ After ten days of growth, metabolites were extracted with 50 mL ethyl acetate. Five drops of HCl were added to 50 mL of ethyl acetate and the agar culture mixture to acidify the extract. The mixture was stirred at room temperature for ten minutes and then anhydrous magnesium sulfate was added to absorb the remaining water. The mixture was filtered and then dried *via* a SpeedVac (using a low boiling point setting). The dried metabolite extract was then dissolved in a 50 : 50 acetonitrile:water mixture. LC-HRMS analyses were carried out with 5 µL of prepared extracts injected through a reverse phase column (Zorbax Eclipse Plus C18, size 2.1 × 100 mm, particle size 1.8 µm) connected to a Dionex 3000RS UHPLC coupled to a Bruker Ultra High Resolution (UHR) Q-TOF MS MaXis II mass spectrometer with an electrospray source. Sodium formate (10 mM) was used for internal calibration and a *m*/*z* scan range of 50–2500 was used. The mobile phase was a gradient mixture of acetonitrile and formic acid in water following the elution programme reported in Table S11.

### DNA purification

The MagAttract HMW DNA Kit (Qiagen) was used for extraction and purification of high-molecular weight genomic DNA of *S. sclerotialus* NRRL ISP-5269, following the manufacturer's instructions.

### Nanopore sequencing

A MinION Flow Cell (FLO-MIN112), native barcoding expansion (EXP-NBD104), native barcoding kit (EXPNBD114), and protocol SQK-LSK109 from ONT were used to perform Nanopore sequencing of the genome of *S. sclerotialus* NRRL ISP-5269, following the manufacturer's instructions.

### Genome sequencing and assembly

The basecalling process was carried out on the raw data using Guppy (Guppy version: 5.0.7 + 2332e8d65).^25^ Assembly of the genome was performed *via* flye (flye version: 2.8.2-b1689).^26^ The assembled data was polished using minimap2 (version: 2.11-r797),^27^ racon (v1.4.20)^28^ and Medaka (version: 1.2.1).^29^ A quality check of the polished data was then carried out using BUSCO (v5.0.0).^30^ The structural and functional annotation was performed with PROKKA (1.14.6).^31^

### Total RNA extraction from *S. albidoflavus*

The Monarch Total RNA Miniprep Kit (New England Biolabs) was used to purify RNA from *S. albidoflavus*/*scl* and *S. albidoflavus*/*scl* Δ*sclM4*, following the manufacturer's instructions.

### Whole transcriptome sequencing and analysis

Extracted total RNA was sequenced by Genewiz Azenta using Illumina NovaSeq 2 × 150 bp sequencing, 10M read pairs, value package.

Galaxy (https://usegalaxy.org. Galaxy Version 1.14.6 + galaxy1) was used for whole transcriptomics analysis.^32^ Trim Galore! was performed for trimming adapters from sequence data.^33^ FastQC was then used for quality control.^34^ The sequence of the heterologously expressed plasmid pCAP03-*scl* was added at the end of the *S. albidoflavus* genome (GenBankCP04370) sequence to reflect insertion of the *scl* gene cluster in the heterologous host, and the combined sequence was used as the reference genome. Bowtie2 was used to align the paired-end, trimmed RNA sequencing reads with the reference genome, with very sensitive local (–very-sensitive-local) parameter presets.^35,36^ FeatureCounts was performed to align the alignments from Bowtie2 with annotated reference genome, with both multi-mapping and multi-overlapping features included (–M –O).^37^ DESeq2 was finally used to normalise the counts of each gene from each sample for the final data analysis.^38^

## Author contributions

J. Z., F. A. and C. C. were responsible for project conceptualisation, experimental design and analysis, with contributions from M. T. J. Z. performed experimental work, analysed and interpreted the data. L. S. performed mass spectrometry analysis. J. Z. wrote the initial manuscript. F.A. reviewed and edited the manuscript with contributions from C. C. All authors read and approved the final manuscript.

## Conflicts of interest

The authors have no competing interests to declare.

## Supplementary Material

CB-OLF-D6CB00137H-s001

## Data Availability

The data supporting this article have been included as part of the supplementary information (SI). Supplementary information: Fig. S1–S8 and Tables S1–S11, oligonucleotides sequences, DNA/RNA sequencing data, gel electrophoresis images, homology search results, bioinformatics data, LC-HRMS conditions. The genome sequence was deposited at DDBJ/ENA/GenBank under the accession JBFOHP000000000. Raw RNA sequencing data was deposited in the NCBI sequence read archive (SRA) under BioProject accession PRJNA1474967 and SRA accessions SRX33758471-6. See DOI: https://doi.org/10.1039/d6cb00137h.
