# Supplementary material for: ArpA-like regulatory control and discovery of new biosynthetic genes driving scleric acid biosynthesis
Source: RSC Chem Biol. 2026 Jun 26;7(8):1642–9. doi: 10.1039/d6cb00137h (PMC13307308; doi:10.1039/d6cb00137h)
Supplement: Supplementary file 1 [file CB-007-D6CB00137H-s001.pdf]

Supplementary Information (SI) for RSC Chemical Biology.
This journal is © The Royal Society of Chemistry 2026

**ArpA-like regulatory control and discovery of new biosynthetic genes driving scleric acid biosynthesis**

Jingfan Zhang,[1] Lijiang Song,[2] Manuela Tosin,[2] Christophe Corre,[1,2*] Fabrizio Alberti[1*]

[1] School of Life Sciences, University of Warwick, Coventry, CV4 7AL, UK.

[2] Department of Chemistry, University of Warwick, Coventry, CV4 7AL, UK.

*E-mail for correspondence: C.Corre@warwick.ac.uk; F.Alberti@warwick.ac.uk.

## Table of content

**Table S1.** Oligonucleotides used for *sclG* knockout. .................................................... 3

**Figure S1.** PCR screening for *sclG* knockout. .............................................................. 5

**Figure S2.** Bandage result for the consensus.fasta file (generated through Medaka) from nanopore sequencing of *S. sclerotialus* (1). .............................................................. 6

**Figure S3.** Quality check using BUSCO........................................................................ 6

**Table S2.** General features of the chromosome............................................................. 6

**Figure S4.** Distribution of the 36 regions predicted to produce secondary metabolites in the *S. sclerotialus* genome........................................................................................... 6

**Table S3.** Identified secondary metabolite regions on *S. sclerotialus* genome by antiSMASH using strictness set as relaxed. .............................................................. 7

**Table S4.** Homology search performed on proteins predicted to be produced by genes in region 21 ........................................................................................................... 9

**Table S5.** Locus tags of SclC and SclK. ....................................................................... 10

**Table S6.** NCBI multiple sequence alignment result for DNA sequence of *sclC* and *sclK* against the *S. albidoflavus* genome ........................................................................... 10

**Table S7.** Normalised counts of each gene on the *scl* gene cluster. ............................. 10

**Table S8.** log2(FC) of genes from the *scl* gene cluster from strain *S. albidoflavus*/*scl* △*sclM4* compared to *S. albidoflavus*/*scl*. ...................................................................... 11

**Figure S5.** Genes differentially transcribed in *S. albidoflavus*/*scl* △*sclM4* compared to *S. albidoflavus*/*scl*. ......................................................................................................... 13

**Figure S6.** IGV visualisation of RNA reads covering the *sclM5* region (21,876-23,012 bp) from *S. albidoflavus*/*scl* Δ*sclM4* strain (top) and *S. albidoflavus*/*scl* strain (bottom). ...... 14

**Figure S7.** The visualized transcriptome map spanning the entire *sclM4* gene, with the 20-bp deletion of *sclM4* in *S. albidoflavus*/*scl* Δ*sclM4* clearly observable in the RNA sequence data. ....................................................................................................................... 14

**Table S9.** Consensus ARE motif sequences for *S. sclerotialus* NRRL ISP-5269 and *S. coelicolor* A3(2) predicted by MEME. ................................................................... 15

**Figure S8.** Visualisation of promoter region on the *scl* gene cluster *via* IGV. ………..16

**Table S10.** List of primers used....................................................................................... 17

**Table S11.** LC-HRMS conditions used for detection of scleric acid and *L*-proline-oxyacetic acid. ........................................................................................................... 17

**Supplementary references** ........................................................................................... 17

**Table S1.** Oligonucleotides used for *sclG* knockout.

| Name | Sequence |
|---|---|
| Protospacer | GCCCACGAGACTACGCTGTT |
| Left homology region | AGTGCGTCGGCGACGACGGCGAGCTGGACGGCCTCGGTGCCGCCTACACCACGCTGTGCGACCCCGGCTCAACCTTCAGCAGTCCATCGCCATCGCCAATACCTGGCCCTGCTAACGGAGAGGTGTCCTTCCGTCCATGGGAATCCCCGCCATCCAGCCCTACCCCATGCCCTCGGCAGCGGACCTTCCGGCCGGCCCTGCCGACTGGAAAGTCGACCCCGAGCGGGCCGTACTTCTCATCCACGACATGCAGAAGTACTTCGTCGATTCCTTCCCCAAGGATTCCGAGCCGGTGACCTCCCTCACCCGGAACGTCCGTGAACTGCGGGAACACTGCGTCAAGCACGCGATCCCGGTGACCTACACCGCGCAGCCCGGCAGCATGAGCGACCGGGACCGCGGCCTGCTGAAGGACTTCTGGGGCCCGGGAATGACGGTGAGCCCCGCCCAGCGGGAGATCGTCCCGGGCATCGAGCCCGAGGGCGGCGACCAGGTGTTCACCAAATGGCGGTACAGCGCCTTCCACCGCACCGGTCTGCTCGACTTCCTGCGCTCGCACGGCCGCGACCAGCTGATCATCTGCGGCATCTACGCCCATATCGGCTGCCTGGCCACCGCCATCGAGTCCTACACGAACGACGTGGAGACGTTCTTCGTGTCCGACGCGGTCGCCGACTTCACCCCCGAACAACACCGCATGGCCCTGGAGTACACGGCCGGACGCTGCGCGGTGGTCCTGCCCACCCGGACGGTCGTGACCCAGATCGAGACGGCCCCGACGACAGTGGAGACCCGATGA |
| Right homology region | CAAGTAAGCGACGAAAGAGCCAAGAGTATGAGTTACAGGAAGATCCTGAGGGGCAATGTCCTGCTCTGGATGATCACCGCATTCTCAGGCCGGCTTCCCATCGCCATCGCTCCGTTGGGCCTCGTCTTCCTGGTCCGGGAGACGCCCGGGGGGTATTCGCTGGGCGCCACGCTGGCCGGTGCCTACGTACTGGGAGAG |

| | |
|---|---|
| | GTGCTCGGGTCAGTGGCGCTCGGCGCCTGGCTGCGTCCCC<br>GGCGGATGAACCTGCACCTCGCTGCCGGGATGGCCGTCG<br>GCGCCCTCGCCTTCGCCGGACTCGCCGTTTTCCCCGACAC<br>CTCGGTGGCGCTCACCAGCACCCTGGCGTTCCTCGCAGGC<br>GCGGCGCCGGCTGCGAGCCCCGGCGGCATGCGCACCATG<br>CTGATCGCCCTGGTCGACAAGGCGGACGAGGCGCGCACG<br>TTGAGCGCCGAGACGGTACTCACCCAGGTCACGTGGGGT<br>GGTGCGCCCGCGCTCGTCGTCGTCCTCGCGGTGAATGCCT<br>GGCCCGGAGCGCCGATGGTGCTCGGCGCGGTGGGATTCG<br>CGGTCGCCGCGGCACTGCTGTTCGTGCTGCCGGCCCACCG<br>GGCCGCGGCCCCGGAGGACGTGGCCGGGGCCGCCGGGGG<br>CCGGGGCAAGGAGCGGGGCAAGCTCCTCGCCTCCGCCTG<br>GCCGATCTACCTCACCAGTGCGGCGGCGATGTCCATGCTC<br>GCCACGGCCGAGCTGGCCCTCACCCCGCTCCTCGAGTACC<br>GCGGCCTGACGGTCAACTGGTCGGGTGTCCTGCTCGCCCT<br>CTTCTCGCT |

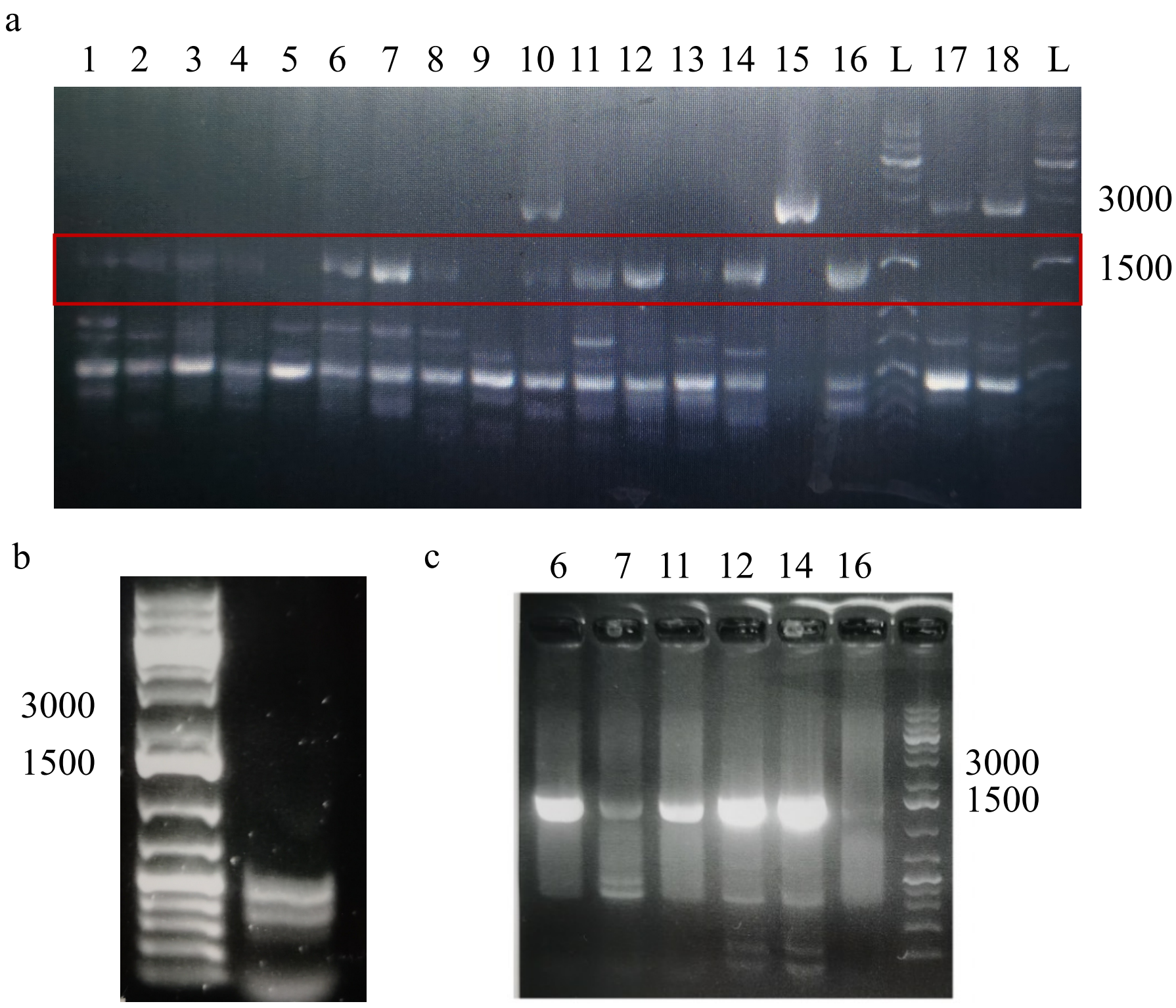


**Figure S1.** PCR screening for *sclG* knockout. For wild type *scl* gene cluster, the PCR results is expected to be 2,972 bp and for the *sclG* knockouts it is expected to be 1,654 bp. (a) Lane 1-16 are colony PCRs of the 16 exconjugants from the conjugation between *S. albidoflavus/scl* Δ*SclM4* and *E. coli* ET12567/pUZ8002 with pCm2-sgRNA-HRs. Lanes 17 and 18 are colony PCRs from *S. albidoflavus/scl* Δ*SclM4*. (b) Colony PCR results from *S. albidoflavus* WT as a control. (c) Lanes 6, 7, 11, 12, 14 and 16 show amplicons from DNA extracted from the same strains as those with the corresponding numbers in panel (a).

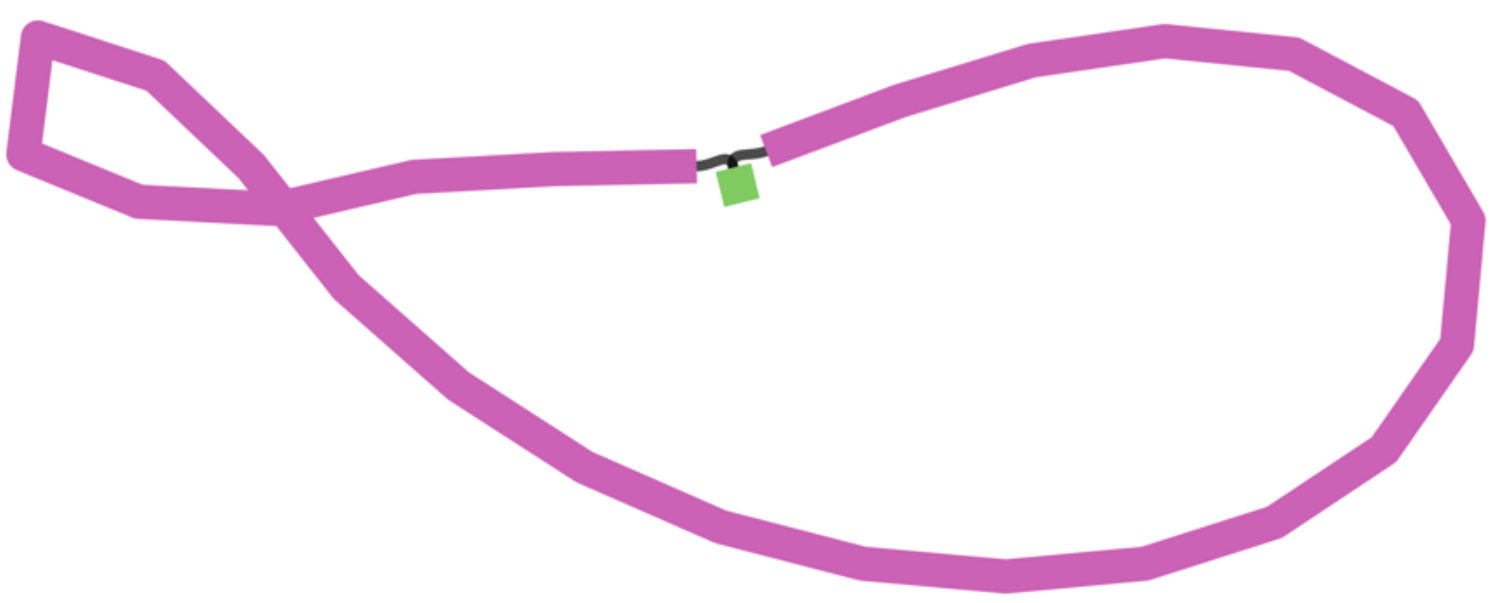

**Figure S2.** Bandage result for the consensus.fasta file (generated through Medaka) from nanopore sequencing of *S. sclerotialus* (1). The result shows that there is a single chromosome with no plasmids. There are two contigs from the sequencing, which are represented in different coloured nodes here.

```
---------------------------------------------------
|Results from dataset streptomycetales_odb10      |
---------------------------------------------------
|C:99.5%[S:98.7%,D:0.8%],F:0.3%,M:0.2%,n:1579     |
|1572   Complete BUSCOs (C)                       |
|1559   Complete and single-copy BUSCOs (S)       |
|13     Complete and duplicated BUSCOs (D)        |
|4      Fragmented BUSCOs (F)                     |
|3      Missing BUSCOs (M)                        |
|1579   Total BUSCO groups searched               |
---------------------------------------------------
```

**Figure S3.** Quality check using BUSCO.

**Table S2.** General features of the chromosome.

| **Component of chromosome** | **Property** |
|---|---|
| Chromosome | 1 |
| Plasmid | 0 |
| Total size | 8,261,949 bp |
| G+C content | 71.84% |
| Coding sequences | 7,175 |
| Ribosomal RNAs | 4 × (5S-23S-16S), 2 × (16S-23S-5S) |
| Transfer RNAs | 69 |
| Contigs | 2 |

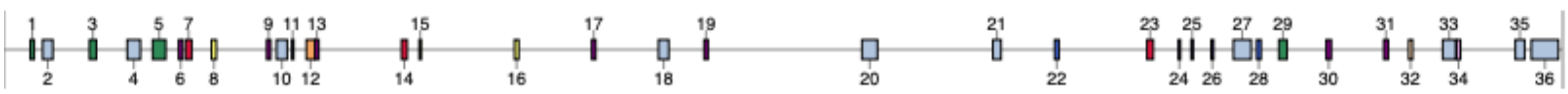


**Figure S4.** Distribution of the 36 regions predicted to produce secondary metabolites in the *S. sclerotialus* genome. Predicted by antiSMASH, with detection strictness set as relaxed. Among them, region 21 contains the *scl* biosynthetic gene cluster (2).

**Table S3.** Identified secondary metabolite regions on *S. sclerotialus* genome by antiSMASH using strictness set as relaxed.

| **Region** | **Type** | **Most similar known cluster (Type)** | **Similarity /%** |
|---|---|---|---|
| 1 | CDPS (tRNA-dependent cyclodipeptide synthases) | Totopotensamide A/totopotensamide B (NRP+Polyketide (3)) | 10 |
| 2 | Thioamide-NRP, NRPS, terpene | 2-methylisoborneol (Terpene (4)) | 100 |
| 3 | NAPAA (Non-alpha poly-amino acids like e-Polylysin) | Stenothricin (NRP: Cyclic depsopeptide (5)) | 13 |
| 4 | Lanthipeptide-class-III, NRP-metallophore, NRPS | Griseobactin (NRP (6)) | 61 |
| 5 | NRPS, NRPS-like | JBIR-126 (NRP (7)) | 89 |
| 6 | Terpene | A201a (Nucleoside (8)) | 5 |
| 7 | NI-siderophore | Peucechelin (NRP (9)) | 25 |
| 8 | Lanthipeptide-class-I | Griselimycin (NRP (10)) | 11 |
| 9 | Terpene | Pristinol (Terpene) | 100 |
| 10 | T1PKS, NRPS | Lankamycin (Polyketide (11)) | 16 |
| 11 | RiPP-like | Hexacosalactone A (Polyene macrolide (12)) | 4 |
| 12 | hglE-KS | Deoxyhangtaimycin (Polyketide+NRP (13)) | 24 |
| 13 | Terpene | / | / |
| 14 | NI-siderophore | Legonoxamine A/ desferrioxamine (Siderophore (14)) | 100 |

| | | | |
|---|---|---|---|
| 15 | Ectoine | Ectoine (Ectoine (15)) | 100 |
| 16 | Lanthipeptide-class-III | Amfs (RiPP:Lanthipeptide (16)) | 60 |
| 17 | Terpene | Neomediomycin B (Polyketide + other) | 7 |
| 18 | Betalactone, NRPS-like | Cosmomycin C (Polyketide (17)) | 5 |
| 19 | Terpene | Tetrachlorizine (Polyketide) | 9 |
| 20 | NRPS, oligosaccharide, other | Pulvomycin B/ pulvomycin C/ pulvomycin D (Polyketide (18)) | 8 |
| 21 | NRPS, butyrolactone | **Scleric acid** (NRP (19)) | 100 |
| 22 | Crocagin | Massinidine (Alkaloid (20)) | 66 |
| 23 | NI-siderophore | Kinamycin (Polyketide) | 19 |
| 24 | Butyrolactone | / | / |
| 25 | RiPP-like | / | / |
| 26 | Hydrogen-cyanide | Aborycin (RiPP (21)) | 14 |
| 27 | Terpene, NRPS, transAT-PKS | Anthracimycin (Polyketide (22)) | 22 |
| 28 | Redox-cofactor | Kanamycin (Saccharide) | 5 |
| 29 | NRPS-like | / | / |
| 30 | Terpene | Hopene (Terpene (23)) | 76 |
| 31 | Terpene | Carotenoid (Terpene (24)) | 45 |
| 32 | Indole | Pentalenolactone (Terpene (25)) | 15 |
| 33 | T1PKS, other, lamthipeptide-class-III | Chlorizidine A (NRP+ Polyketide: Modular type 1 polyketide (26)) | 11 |
| 34 | Phenazine | Meilingmycin (Polyketide (27)) | 4 |
| 35 | NRPS, T1PKS | Griseoviridin/ fijimycin A (NRP: Cyclic depsipeptide: Trans-AT type 1 polyketide (28, 29)) | 5 |

| | | | |
|---|---|---|---|
| 36 | NRPS, NRPS-like, T2PKS, RiPP-like, T1PKS | Limazepine C/ limazepine D/ limazepine E/ limazepine F/ limazepine A (NRP (30)) | 100 |

**Table S4.** Homology search performed on proteins predicted to be produced by genes in region 21 (identified by antiSMASH as containing the *scl* gene cluster, as reported in Table S3). SclC-SYN_04643 are upstream of *sclE* whereas SYN_04668-SYN_04673 are downstream of SclP. Apart from SclC and SclK, all the other proteins do not appear to be involved in specialised metabolite biosynthesis and thus do not seem to belong to the *scl* cluster.

| Name | Putative function |
|---|---|
| SYN_04643 | Hypothetical protein |
| SYN_04644 | DUF6507 family protein |
| SYN_04645 | DEAD/DEAH box helicase family protein |
| SYN_04646 | Type I restriction-modification system |
| SYN_04647 | Type I restriction-modification system subunit M |
| SclK | glutamate 5-kinase |
| SclC | chorismate synthase |
| SYN_04650-SYN_04667 | *scl* original cluster |
| SYN_04668 | DUF6177 family protein |
| SYN_04669 | Type VII secretion protein EccCa |
| SYN_04670 | ATPase |
| SYN_04671 | Type VII secretion integral membrane protein EccD |
| SYN_04672 | Hypothetical protein |
| SYN_04673 | DUF6508 domain-containing protein |

**Table S5.** Locus tags of SclC and SclK.

| **Gene** | **Locus tag** |
|---|---|
| *sclC* | AAC944_RS23385 |
| *sclK* | AAC944_RS23380 |

**Table S6.** NCBI multiple sequence alignment result for DNA sequence of *sclC* and *sclK* against the *S. albidoflavus* genome (with *Actinomyces albidoflavus* (taxid:1886) as search organism), showing that homologues of *sclC* and *sclK* are present in *S. albidoflavus*.

| **Protein** | **Homology** | **Identity** |
|---|---|---|
| SclC | Chorismate synthase | 84.39% |
| SclK | Glutamate 5-kinase | 81.25% |

**Table S7.** Normalised counts of each gene on the *scl* gene cluster. *scl*/1,2,3 are triplicates of *S. albidoflavus*/*scl* whereas ∆*sclM4*/1,2,3 are triplicates of *S. albidoflavus*/*scl* ∆*sclM4*. *Reference 1* (*XNR_4871*) and *Reference 2* (*XNR_5311*) are orthologs of two selected reference genes from *Streptomyces coelicolor* (31).

| **Genes(*scl*)** | ***scl*/1** | ***scl*/2** | ***scl*/3** | **∆*sclM4*/1** | **∆*sclM4*/2** | **∆*sclM4*/3** |
|---|---|---|---|---|---|---|
| *P* | 86.90 | 80.21 | 73.18 | 1639.50 | 4451.67 | 2689.19 |
| *Q4* | 207.09 | 103.80 | 147.18 | 4307.64 | 10568.94 | 6982.86 |
| *Q3* | 171.96 | 77.51 | 155.31 | 4767.37 | 11041.99 | 7643.87 |

| | | | | | | |
|---|---|---|---|---|---|---|
| *Q2* | 192.30 | 114.59 | 162.63 | 5873.55 | 14740.75 | 9413.90 |
| *Q1* | 59.17 | 26.96 | 49.60 | 1603.10 | 3929.54 | 2347.69 |
| *M1* | 127.58 | 80.21 | 97.58 | 873.56 | 1178.97 | 963.15 |
| *M2* | 332.83 | 264.89 | 322.81 | 5027.69 | 7634.57 | 6015.08 |
| *L* | 123.89 | 123.35 | 148.80 | 1939.39 | 2670.17 | 1939.04 |
| *M3* | 170.11 | 370.72 | 234.18 | 1137.84 | 2151.17 | 1362.54 |
| *M5* | 225.58 | 561.47 | 282.97 | 685.23 | 1555.95 | 990.94 |
| *N* | 645.31 | 433.40 | 593.59 | 6299.25 | 17304.41 | 11217.49 |
| *T* | 155.32 | 72.80 | 120.34 | 1606.27 | 4264.75 | 2740.12 |
| *A* | 253.32 | 124.02 | 215.48 | 2947.46 | 8094.05 | 5119.07 |
| *D* | 186.75 | 124.70 | 162.63 | 1864.22 | 5271.42 | 3506.48 |
| *I* | 131.28 | 101.78 | 152.06 | 2008.23 | 6212.29 | 4338.82 |
| *G* | 469.65 | 711.10 | 760.28 | 2389.62 | 7333.83 | 4813.45 |
| *E* | 1218.51 | 1346.71 | 1310.77 | 1054.76 | 2546.95 | 1717.93 |
| *Reference 1*(*XNR_4871*) | 94.30 | 181.99 | 138.23 | 170.12 | 308.06 | 226.90 |
| *Reference 2*(*XNR_5311*) | 451.164 | 306.01 | 350.46 | 193.068 | 222.427 | 184.064 |

**Table S8.** log2(FC) of genes from the *scl* gene cluster from strain *S. albidoflavus/scl* △*sclM4* compared to *S. albidoflavus/scl*. *Reference 1* (*XNR_4871*) and *Reference 2*(*XNR_5311*) are orthologs of two selected reference genes from *Streptomyces coelicolor* (31).

| **genes (*scl*)** | **log2(FC)** | **P-value** | **P-adj** |
|---|---|---|---|
| *P* | 5.19 | 1.01E-42 | 3.50E-40 |
| *Q4* | 5.58 | 3.59E-46 | 1.76E-43 |
| *Q3* | 5.86 | 3.66E-48 | 2.39E-45 |
| *Q2* | 6.00 | 6.41E-57 | 7.53E-54 |
| *Q1* | 5.86 | 3.39E-45 | 1.53E-42 |

| | | | |
|---|---|---|---|
| *M1* | 3.30 | 2.29E-36 | 5.62E-34 |
| *M2* | 4.34 | 2.17E-69 | 1.28E-65 |
| *L* | 4.05 | 1.12E-61 | 3.28E-58 |
| *M3* | 2.59 | 2.73E-12 | 3.87E-11 |
| *M5* | 1.59 | 0.00017346 | 0.00057658 |
| *N* | 4.38 | 9.09E-31 | 1.15E-28 |
| *T* | 4.63 | 5.32E-29 | 5.79E-27 |
| *A* | 4.77 | 1.56E-30 | 1.91E-28 |
| *D* | 4.49 | 9.22E-31 | 1.15E-28 |
| *I* | 5.03 | 1.18E-34 | 2.24E-32 |
| *G* | 2.90 | 1.26E-12 | 1.86E-11 |
| *E* | 0.46 | 0.17156144 | 0.2431902 |
| *Reference 1*(*XNR_4871*) | 0.78 | 0.02677902 | 0.04978814 |
| *Reference 2*(*XNR_ XNR_5311*) | -0.87 | 0.00029186 | 0.00091773 |

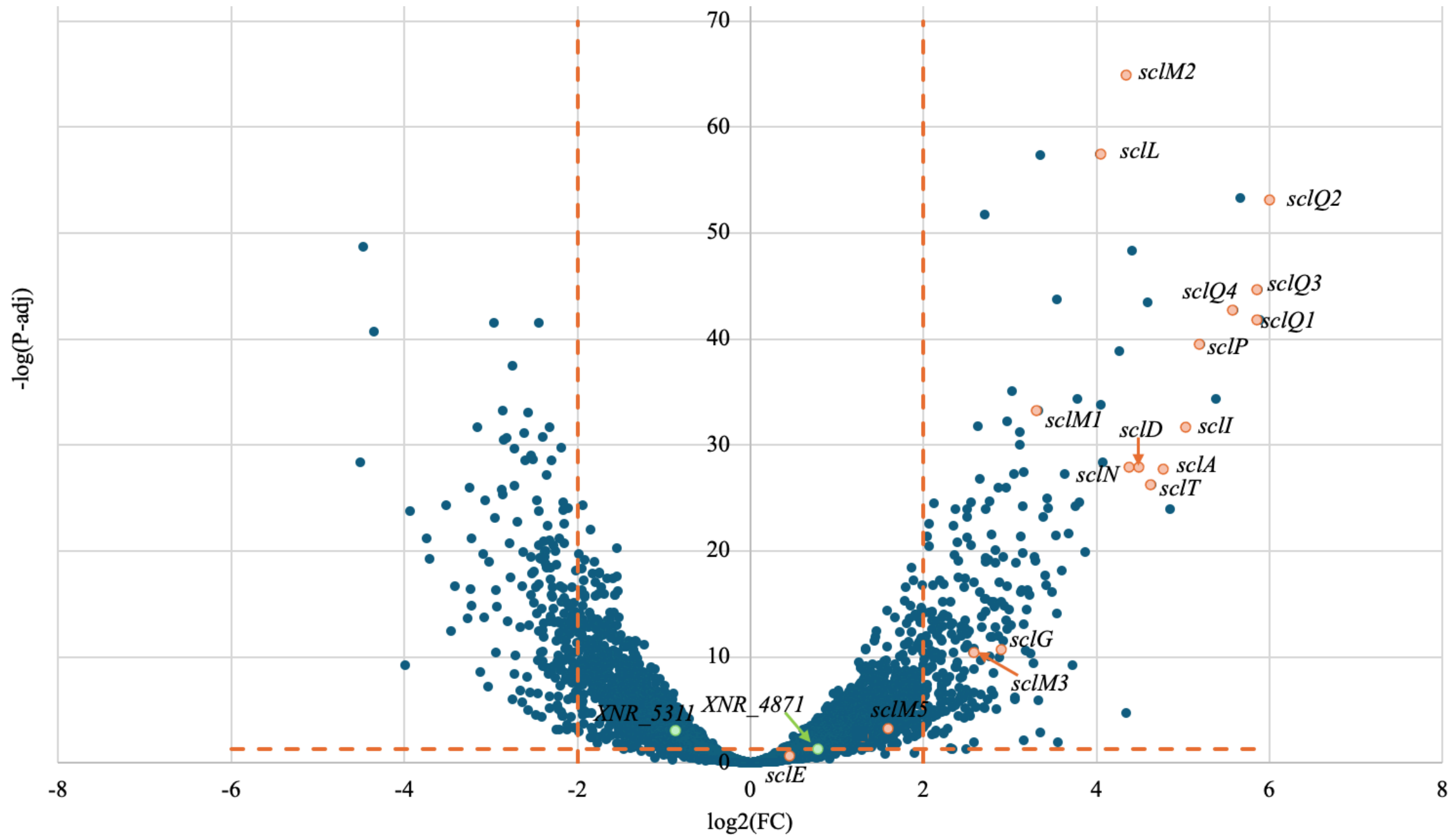


**Figure S5.** Genes differentially transcribed in *S. albidoflavus/scl* Δ*sclM4* compared to *S. albidoflavus/scl*. Orange dots represent the genes from the *scl* gene cluster. Green dots represent two reference genes *XNR-4871* and *XNR_5311*, which are orthologs of two selected reference genes from *Streptomyces coelicolor* coding for ABC-type branched-chain amino acid transport system and Protein-disulfide isomerase, respectively (31). Blue dots represent genes from the *S. albidoflavus* genome and the rest of the genes from the pCAP03 plasmid used to capture the *scl* gene cluster. The orange line shows the cutoff of differentially transcribed genes (|log2Fold|>2 with p-adjust <0.05).

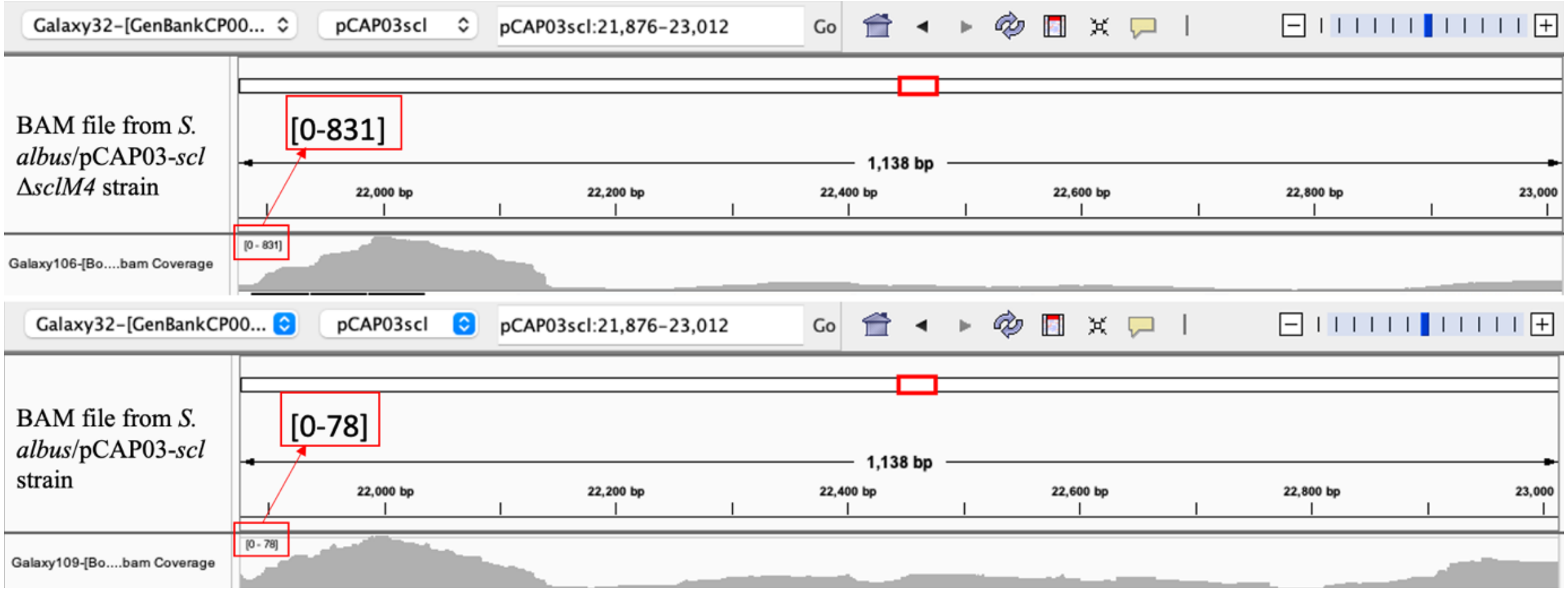


**Figure S6.** IGV visualisation of RNA reads covering the *sclM5* region (21,876-23,012 bp) from *S. albidoflavus*/*scl* Δ*sclM4* strain (top) and *S. albidoflavus*/*scl* strain (bottom). The numbers in the red frame are the read depth of the RNA data.

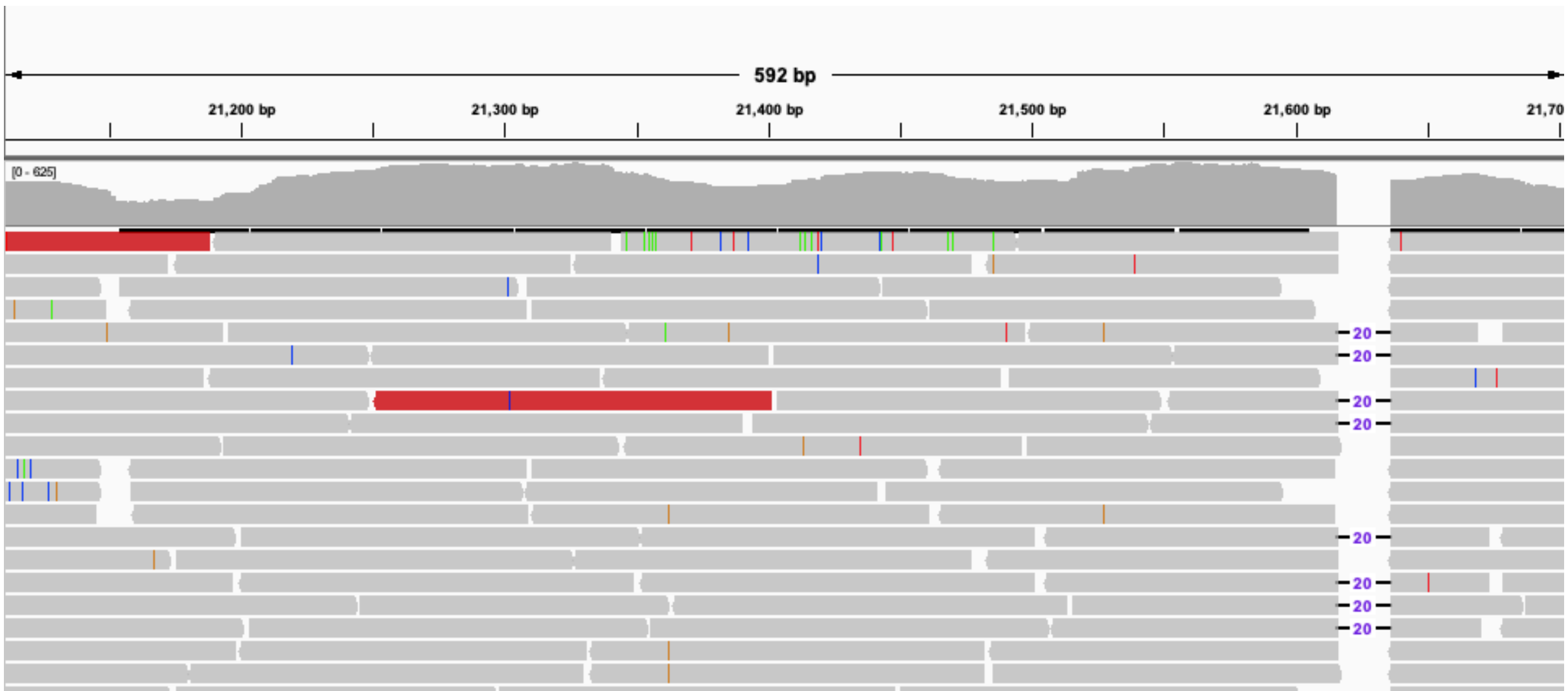


**Figure S7.** The visualized transcriptome map spanning the entire *sclM4* gene, with the 20-bp deletion of *sclM4* in *S. albidoflavus*/*scl* Δ*sclM4* clearly observable in the RNA sequence data.

**Table S9.** Consensus ARE motif sequences for *S. sclerotialus* NRRL ISP-5269 and *S. coelicolor* A3(2) predicted by MEME. The consensus ARE motifs in both strains are highly similar. The table includes predicted sites from *S. sclerotialus*.

<u>*S. sclerotialus* NRRL ISP-5269</u>

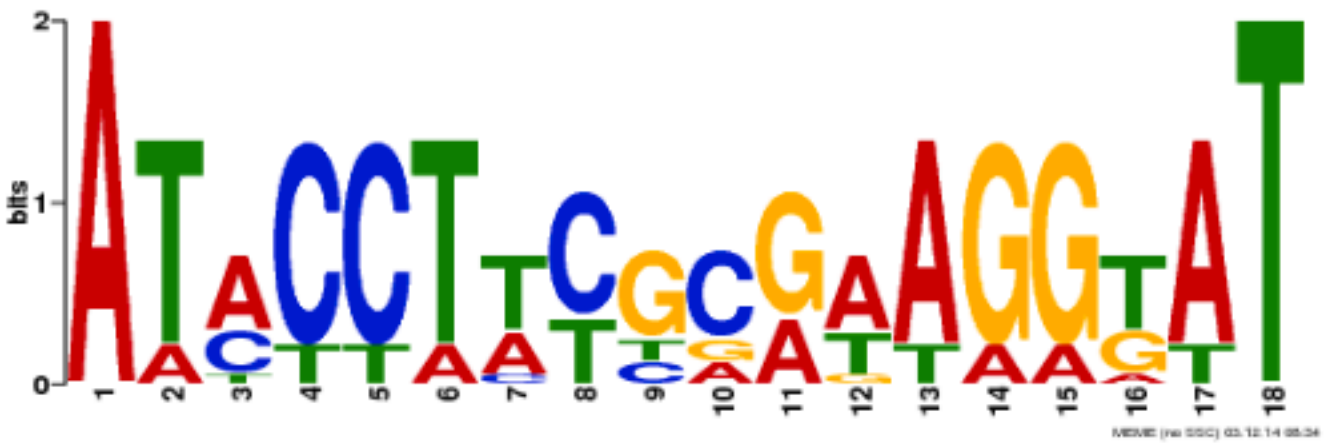

E-value: 9.0e-004

<u>*S. coelicolor* A3(2)</u>

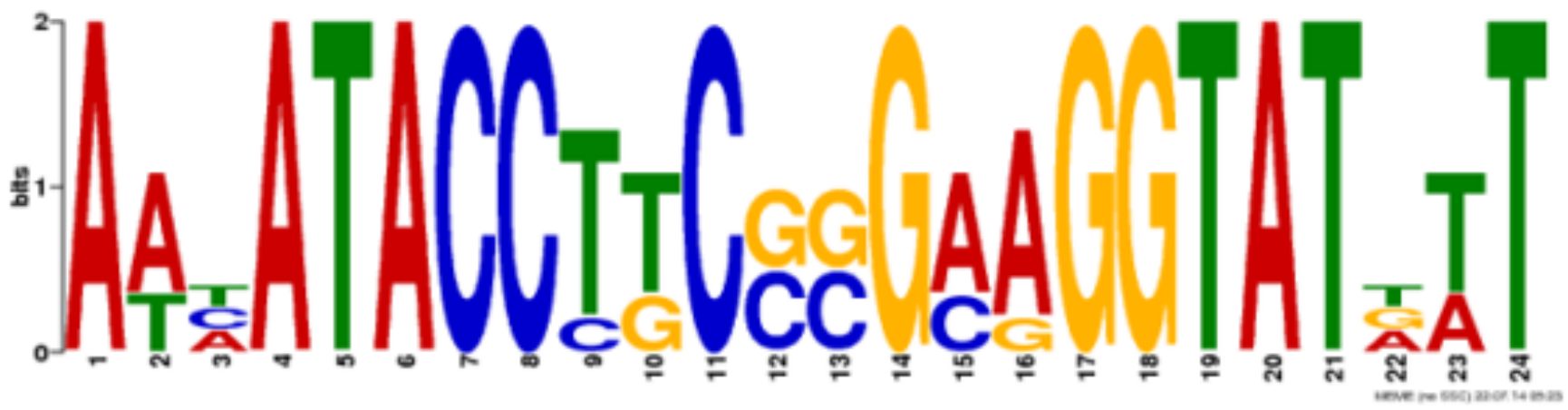

E-value: 5.9e-005

| **Gene location** | **Bp upstream of start codon** | ***P*-value** | **Sites (Coloured nucleotides represent the predicted ARE motif and black nucleotides represent sequences flanking the motif)** |
|---|---|---|---|
| Upstream of *sclM1* | 171 | 5.43e-10 | GCGAAAAAATATACCTATGGGAAGGTATATTGCATGGG |
| Upstream of *sclM2* | 162 | 6.96e-10 | CCCATGCAATATACCTTCCCATAGGTATATTTTTTCGC |
| Upstream of *sclQ1* | 114 | 3.19e-9 | ATCTCAATTGAAACCTTCGCGAAGGTTTGATTCTGTGG |
| Upstream of *sclN* | 43 | 1.25e-8 | TGACATTTGAATACCTCCGCGAAGGAATGATTTCGTGA |
| Upstream of *sclM4* | 52 | 1.59e-7 | TGTGTTGGGCATCCCAATGAGAAAAGATCCGCCTTGGA |
| Upstream of *sclM5* | 104 | 1.75e-7 | TCCAAGGCGGATCTTTTCTCATTGGGATGCCCAACACA |

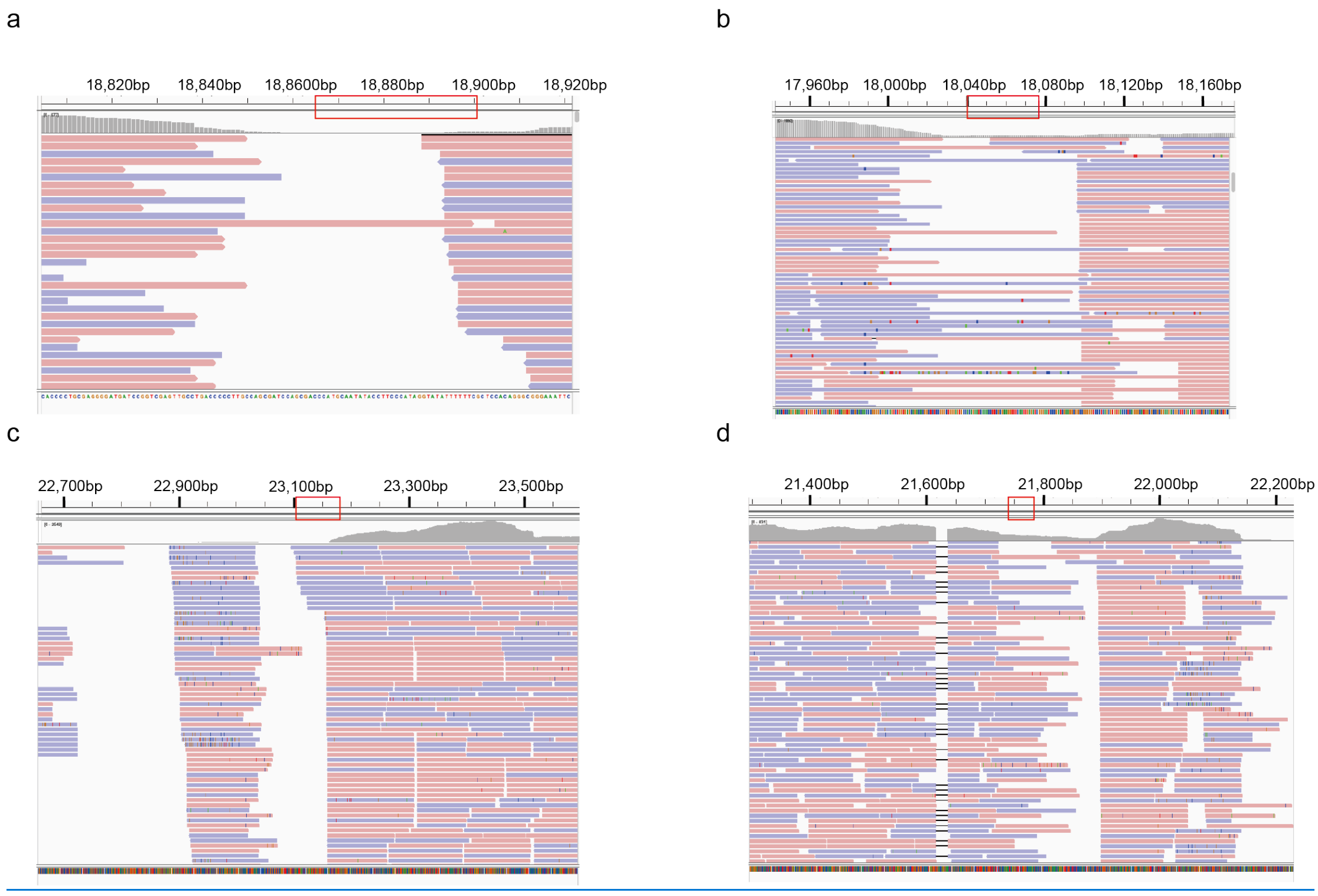


**Figure S8.** Visualisation of promoter region on the *scl* gene cluster *via* IGV. Predicted ARE sequences are framed in red. (a) Promoter region between *sclM1* (gene location: 18,125-18,703 bp) and *sclM2* (gene location: 19,055-19,972 bp) (ARE sequence was found in between 18,865-18,902 bp). (b) Promoter region upstream of *sclQ* (gene location: 16,785-17,702 bp) (ARE sequence was found in between 18,040-18,077 bp). (c) Promoter region upstream of *sclN* (gene location: 23,182-26,433 bp) (ARE sequence was found in between 23,111-23,148 bp). (d) Promoter region between *sclM4* (gene location: 21,111-21,701 bp) and *sclM5* (gene location: 21,876-23,012 bp) (ARE sequence was found in between 21,744-21,781 bp).(32) Colours represent alignment of different strands, pink represent forward and purple reverse.

**Table S10.** List of primers used. In instances where there are orange and black colours, orange coloration denotes overhang regions whereas black denotes directly annealing bases.

| Primers | Primer sequence (5'-3') | Notes |
|---|---|---|
| pCm2-*sclG* forward | TCGGTTGCCGCCGGGCGTTTTTTATAGTGCGTCGGCGACGACGG | Screen for *sclG* knockout |
| pCm2-*sclG* reverse | GCGGCCTTTTTACGGTTCCTGGCCTAGCGAGAAGAGGGCGAG | Screen for *sclG* knockout |
| pCm2-*sclG scl* FF | TGCATCTGGAAGCCACCG | Sequencing for *sclG* knockout |
| pCm2-*sclG scl* RR | AAGGCCCCGAAGGCGCTC | Sequencing for *sclG* knockout |

**Table S11.** LC-HRMS conditions used for detection of scleric acid and *L*-proline-oxyacetic acid.

| Retention time (min) | Flow (mL/min) | Acetonitrile (%) | Water (%) |
|---|---|---|---|
| 0 | 0.2 | 5 | 95 |
| 5 | | 5 | 95 |
| 17.3 | | 100 | 0 |
| 22.3 | | 100 | 0 |
| 25.3 | | 5 | 95 |
| 33.3 | | 5 | 95 |

**Supplementary references**


1. Wick RR, Schultz MB, Zobel J, Holt KE. Bandage: interactive visualization of de novo genome assemblies. Bioinformatics. 2015;31(20):3350-2.
2. Blin K, Shaw S, Augustijn HE, Reitz ZL, Biermann F, Alanjary M, et al. antiSMASH 7.0: new and improved predictions for detection, regulation, chemical structures and visualisation. Nucleic Acids Research. 2023;51(W1):W46-W50.
3. Lin Z, Flores M, Forteza I, Henriksen NM, Concepcion GP, Rosenberg G, et al. Totopotensamides, polyketide-cyclic peptide hybrids from a mollusk-associated bacterium Streptomyces sp. J Nat Prod. 2012;75(4):644-9.
4. Jung R, Schaefer V. 13 - Reducing cork taint in wine. In: Reynolds AG, editor. Managing Wine Quality: Woodhead Publishing; 2010. p. 388-417.

5. Liu WT, Lamsa A, Wong WR, Boudreau PD, Kersten R, Peng Y, et al. MS/MS-based networking and peptidogenomics guided genome mining revealed the stenothricin gene cluster in Streptomyces roseosporus. J Antibiot (Tokyo). 2014;67(1):99-104.
6. Patzer SI, Braun V. Gene Cluster Involved in the Biosynthesis of Griseobactin, a Catechol-Peptide Siderophore of *Streptomyces* sp. ATCC 700974. Journal of Bacteriology. 2010;192(2):426-35.
7. Midde S, Vytla D, Velayuthaperumal R, Kaliyaperumal K, Reddy CA, Jarugu LB, et al. Solid-phase synthesis of JBIR-126 (Tambromycin), JBIR-35 and their analogs. Tetrahedron Letters. 2021;69:152970.
8. Saugar I, Sanz E, Rubio MÁ, Espinosa JC, Jiménez A. Identification of a set of genes involved in the biosynthesis of the aminonucleoside moiety of antibiotic A201A from Streptomyces capreolus. European Journal of Biochemistry. 2002;269(22):5527-35.
9. Kodani S, Komaki H, Suzuki M, Kobayakawa F, Hemmi H. Structure determination of a siderophore peucechelin from Streptomyces peucetius. Biometals. 2015;28(5):791-801.
10. Kling A, Lukat P, Almeida DV, Bauer A, Fontaine E, Sordello S, et al. Antibiotics. Targeting DnaN for tuberculosis therapy using novel griselimycins. Science. 2015;348(6239):1106-12.
11. Zhang M, Shuang B, Arakawa K. Accumulation of lankamycin derivative with a branched-chain sugar from a blocked mutant of chalcose biosynthesis in Streptomyces rochei 7434AN4. Bioorganic & Medicinal Chemistry Letters. 2023;80:129125.
12. Duan H, Wang F, Zhang C, Dong Y, Li H, Xiao F, et al. Elucidation of the Late Steps during Hexacosalactone A Biosynthesis in Streptomyces samsunensis OUCT16-12. Applied and Environmental Microbiology. 2023;89(3):e01958-22.
13. Li X, Fu J, Li Y, Liu J, Gao R, Shi Y, et al. Cytochrome P450 Monooxygenase for Catalyzing C-42 Hydroxylation of the Glycine-Derived Fragment in Hangtaimycin Biosynthesis. Org Lett. 2022;24(6):1388-93.
14. Maglangit F, Tong MH, Jaspars M, Kyeremeh K, Deng H. Legonoxamines A-B, two new hydroxamate siderophores from the soil bacterium, Streptomyces sp. MA37. Tetrahedron Letters. 2019;60(1):75-9.
15. Alexander S. Reshetnikov VNK, Ildar I. Mustakhimov, and Yuri A. Trotsenko. Genes and Enzymes of Ectoine Biosynthesis in Halotolerant Methanotrophs. Methods in Enzymology. 2011;495.
16. Ueda K, Oinuma K-I, Ikeda G, Hosono K, Ohnishi Y, Horinouchi S, et al. AmfS, an Extracellular Peptidic Morphogen in *Streptomyces griseus*. Journal of Bacteriology. 2002;184(5):1488-92.
17. Kim J, Lee Y-J, Shin D-S, Jeon S-H, Son K-H, Han DC, et al. Cosmomycin C inhibits signal transducer and activator of transcription 3 (STAT3) pathways in MDA-MB-468 breast cancer cell. Bioorganic & Medicinal Chemistry. 2011;19(24):7582-9.
18. Moon K, Cui J, Kim E, Riandi ES, Park SH, Byun WS, et al. Structures and Biosynthetic Pathway of Pulvomycins B–D: 22-Membered Macrolides from an Estuarine Streptomyces sp. Organic Letters. 2020;22(14):5358-62.

19. Alberti F, Leng DJ, Wilkening I, Song LJ, Tosin M, Corre C. Triggering the expression of a silent gene cluster from genetically intractable bacteria results in scleric acid discovery. Chemical Science. 2019;10(2):453-63.
20. Lombe BK, Winand L, Diettrich J, Töbermann M, Hiller W, Kaiser M, et al. Discovery, Biosynthetic Origin, and Heterologous Production of Massinidine, an Antiplasmodial Alkaloid. Organic Letters. 2022;24(15):2935-9.
21. Shao M, Ma J, Li Q, Ju J. Identification of the Anti-Infective Aborycin Biosynthetic Gene Cluster from Deep-Sea-Derived Streptomyces sp. SCSIO ZS0098 Enables Production in a Heterologous Host. Mar Drugs. 2019;17(2).
22. Hensler ME, Jang KH, Thienphrapa W, Vuong L, Tran DN, Soubih E, et al. Anthracimycin activity against contemporary methicillin-resistant Staphylococcus aureus. J Antibiot (Tokyo). 2014;67(8):549-53.
23. Siedenburg G, Jendrossek D. Squalene-Hopene Cyclases. Applied and Environmental Microbiology. 2011;77(12):3905-15.
24. Davis H, Su X, Shen Y, Xu J, Wang D, Scott Smith J, et al. Chapter 8 - Phenotypic Diversity of Colored Phytochemicals in Sorghum Accessions With Various Pericarp Pigments. In: Watson RR, editor. Polyphenols in Plants (Second Edition): Academic Press; 2019. p. 123-31.
25. Cane DE. 2.06 - Sesquiterpene Biosynthesis: Cyclization Mechanisms. In: Barton SD, Nakanishi K, Meth-Cohn O, editors. Comprehensive Natural Products Chemistry. Oxford: Pergamon; 1999. p. 155-200.
26. Alvarez-Mico X, Jensen PR, Fenical W, Hughes CC. Chlorizidine, a cytotoxic 5H-pyrrolo[2,1-a]isoindol-5-one-containing alkaloid from a marine Streptomyces sp. Org Lett. 2013;15(5):988-91.
27. Sun Y, Zhou X, Tu G, Deng Z. Identification of a gene cluster encoding meilingmycin biosynthesis among multiple polyketide synthase contigs isolated from Streptomyces nanchangensis NS3226. Arch Microbiol. 2003;180(2):101-7.
28. Sun P, Maloney KN, Nam S-J, Haste NM, Raju R, Aalbersberg W, et al. Fijimycins A–C, three antibacterial etamycin-class depsipeptides from a marine-derived Streptomyces sp. Bioorganic & Medicinal Chemistry. 2011;19(22):6557-62.
29. Barbacid M, Contreras A, Vazquez D. The mode of action of griseoviridin at the ribosome level. Biochimica et Biophysica Acta (BBA) - Nucleic Acids and Protein Synthesis. 1975;395(3):347-54.
30. Fotso S, Zabriskie TM, Proteau PJ, Flatt PM, Santosa DA, Sulastri, et al. Limazepines A−F, Pyrrolo[1,4]benzodiazepine Antibiotics from an Indonesian Micrococcus sp. Journal of Natural Products. 2009;72(4):690-5.
31. Li S, Wang W, Li X, Fan K, Yang K. Genome-wide identification and characterization of reference genes with different transcript abundances for Streptomyces coelicolor. Scientific Reports. 2015;5(1):15840.
32. Poon V. Analysis and exploitation of AHFCA-dependent signalling systems in Streptomyces bacteria. University of Warwick: University of Warwick; 2015.
